# Use of genotyping based clustering to quantify recent tuberculosis transmission in Guadeloupe during a seven years period: analysis of risk factors and access to health care

**DOI:** 10.1186/1471-2334-13-364

**Published:** 2013-08-02

**Authors:** Séverine Ferdinand, Julie Millet, Annick Accipe, Sylvie Cassadou, Pascal Chaud, Maryse Levy, Max Théodore, Nalin Rastogi

**Affiliations:** 1 Observatoire Régional de la Santé de la Guadeloupe, Basse-Terre, Guadeloupe, France; 2 WHO Supranational TB Reference Laboratory, Institut Pasteur de la Guadeloupe, Abymes, Guadeloupe, France; 3 Direction des Actions de Solidarité Départementale, Conseil Général de la Guadeloupe, Basse-Terre, Guadeloupe, France; 4 CIRE Antilles-Guyane –InVS, Gourbeyre, Guadeloupe, France; 5 CIRE Antilles-Guyane –InVS, Fort de France, Martinique, France

**Keywords:** Mycobacterium, Tuberculosis, Transmission, Guadeloupe, Genotyping, Spoligotyping, Exact-Tandem-Repeats, Database, Drug-resistance

## Abstract

**Background:**

The present study aimed to characterize *Mycobacterium tuberculosis* population structure and to identify transmission chains and risk factors by prospective molecular typing in conjunction with conventional epidemiological investigations in the French overseas department of Guadeloupe.

**Methods:**

The study included all the culture-positive TB cases (1 clinical isolate per patient; n = 129) diagnosed between a seven year period (April 4^th^, 1999 to December 31^st^, 2005). Prospective molecular typing was performed using spoligotyping and VNTRs, and a subset of 44 *M. tuberculosis* isolates found to be clustered was retrospectively typed using 12-loci MIRUs. Data were compared using the SITVIT2 database, followed by analysis of risk factors in function of clustering of the isolates and available demographic and socioeconomic data.

**Results:**

The study sample was characterized by a majority of new cases (87.4%); a moderate proportion of drug-resistance (7.8%); a high level of immigration (51.2% foreign-born) originating from high TB/HIV incidence neighboring islands such as Haiti or Dominican Republic; lower socioeconomic conditions (70.7% of jobless, average income 824 EUR/month); and a significantly higher proportion of TB/HIV co-infected cases (38.2% vs. 8.5%; *p < 0.001*), and extrapulmonary disease (18.2% vs. 4.8%; *p < 0.02*) among migrants as compared to French patients. The study revealed an important delay in access to healthcare with a median delay of 74.5 days between the 1st symptoms and clinical suspicion of TB. Prospective molecular typing based on spoligotyping and 5-loci VNTRs showed that evolutionary recent Euro-American lineages predominated in Guadeloupe (91.5% of isolates). In conjunction with epidemiological data, it allowed to estimate a recent transmission rate of 18.6%, which was close to the rate of 16.7% estimated using retrospective 12-loci MIRU typing. Although a higher proportion of cases in older age-group were apparently linked to reactivation; univariate analysis of risk factors did not allow pinpointing specific risk factors for a patient to belong to a TB transmission group.

**Conclusions:**

Ongoing TB transmission in the insular, low TB-incidence setting of Guadeloupe can be defined as follows: (i) a significant proportion of imported cases of the disease from neighboring islands; (ii) significantly higher TB/HIV coinfection among foreign-born cases; and, (iii) a higher proportion of cases affecting older age-group among French patients due to reactivation. This study emphasizes the need for universal typing using spoligotyping and 15-loci MIRUs in prospective studies.

## Background

There has been major progress in reducing tuberculosis (TB) cases in the past two decades, yet the global burden of TB remains enormous. The World Health Organization (WHO) estimated that there were 8.7 million incident cases of TB and 1.4 million deaths in 2011 [1]; HIV pandemic apparently played a major role in it, since 13% of the TB patients were co-infected with HIV and accounted for 30.7% of all deaths due to TB. Even if most of the TB cases occurred in Asia (59%) and Africa (26%), a non negligible proportion was reported in the Eastern Mediterranean (7%) and European (5%) regions, followed by the Americas (3%). To achieve the current goal of a high level of case detection (75%) and cure (85%) by the year 2015, a finer study of the epidemiological characteristics of TB patients to pinpoint risk factors involved and access to health care is needed in different regions of the world [2]. Molecular fingerprinting of *Mycobacterium tuberculosis* has been proposed as an ideal investigational strategy to achieve this objective, since it allows to incorporate genotyping based clustering to quantify recent TB transmission in order to analyze risk factors and access to health care [3-5]. We hereby report results of such an investigational approach under a prospective genotyping study covering TB transmission during a 7 year period (1999–2005) in Guadeloupe, a French overseas department of the Americas. Under this study, all the *M. tuberculosis* strains were characterized by spoligotyping [6] and Variable Number of Tandem DNA Repeats (VNTRs; [7]) that were methods of choice when this investigation was planned back in 1998, with the aim of determining potential transmission links between TB patients. This prospective study also undertook concomitant investigations to study the access to health care and follow-up of the TB patients diagnosed.

## Methods

### Study design, patients, and *M. tuberculosis* isolates

After a prior explanation of the recommended investigations for the diagnostic purposes by a physician, patients were asked to sign an agreement letter and the pathological specimens were obtained from suspected tuberculosis patients. Only patients residing in Guadeloupe for more than three months were eligible. The cultures were performed using Löwenstein-Jensen slants at 37°C, and were duly identified as *M. tuberculosis* complex using classical biochemical tests and the AccuProbe test (GenProbe Inc., San Diego, CA). All positive cultures were also subjected to drug-susceptibility testing using the proportional method on Löwenstein-Jensen media. The study performed between April 4^th^, 1999 and December 31^st^, 2005 corresponded to a recruitment of 129 TB patients with one *M. tuberculosis* clinical isolate per patient. For each patient, the data requested included nature of pathological specimen, result of AFB smear, the date of diagnosis, the clinical form of the disease, results of HIV serology, and previous history of TB. Demographic and socioeconomic data as well as information relating to the diagnosis were collected by the investigator after individually interviewing each patient using a specific form.

### Ethical considerations

The study protocol was presented to the Conseil National de l’Ordre des Médecins (http://www.conseil-national.medecin.fr/qu-est-ce-que-l-ordre-1206), which guarantees the quality of healthcare provided to the patients. As recommended, it was thereafter submitted to the Commission nationale de l’informatique et des libertés (CNIL; http://www.cnil.fr/english/the-cnil/operation/#c1556) which ensures the protection of personal data. Patients were duly informed about the description and aims of the study in writing, and those enrolled after their oral consent were asked to sign an agreement letter, returned to investigators. The study was approved by CNIL under a formal agreement number 999343 (modalities of registration, storage and analysis of epidemiological, clinical and socioeconomic data are available online from: http://www.pasteur-guadeloupe.fr/tb/projects/tuberculose.pdf).

### Molecular typing

Right from the beginning of the prospective study in 1999, the molecular typing was planned using two PCR-based systems - spoligotyping [6] and VNTRs [7], since this typing scheme is significantly cheaper and faster than the historical “gold standard” IS*6110*-RFLP that was used for earlier *M. tuberculosis* genotyping work in Guadeloupe [8-10]; yet provided with results that were relevant for epidemiological investigations around clustered cases of TB [11]. Briefly, the genomic bacterial DNA was prepared by the cetyltrimethylammonium bromide (CTAB) method [12] from cultured strains. Spoligotyping using primers designated DRa and DRb (with DRa biotinylated at 5’) to amplify the whole Direct Repeat (DR) region was performed as described previously [6]. As spoligotyping used alone may overestimate the number of potentially linked isolates, it was used in association with VNTR typing using exact tandem repeats ETR-A to ETR-E as a 2^nd^-line VNTR typing method as reported [7].

After the completion of the study, a subset of 44 *M. tuberculosis* isolates found to be clustered by combination of spoligotyping and 5-loci VNTRs was retrospectively typed by using the 12-loci MIRU-VNTRs system described later [13]; since it allows a better discrimination than the 5-loci VNTRs that were available when the prospective study was planned back in 1998. The PCR-based 12-loci MIRU-VNTR typing was performed using primers described previously [13,14], and the presence and size of each PCR product was determined manually by electrophoresis on an agarose gel, followed by staining with ethidium bromide as reported previously [15]. Note that although theoretically ETR-D and E correspond to MIRU locus 4 and 31, the primers and calculations based on ETR determination according to the protocols of Frothingham and Meeker-O’Connell [7] versus Supply et al. [13,14], might not turn up in identical copy number for ETR-D and MIRU-4.

### Genotype analysis, database comparison, and recent transmission rate

Spoligotype patterns as octal codes, 5-loci VNTRs, and 12-loci MIRU-VNTR data were entered in the SITVIT2 proprietary database of the Institut Pasteur de la Guadeloupe, which is an updated in-house version of the publicly released SpolDB4 [16] and SITVITWEB [17] databases. In this database, Spoligotype International Type (SIT), VNTR International Type (VIT), and MIRU International Type (MIT) designate identical patterns shared by 2 or more patient isolates, whereas “orphan” designates patterns reported for a single isolate that does not correspond to any of the patterns recorded in the repository of the SITVIT2 database. Major phylogenetic clades were assigned according to signatures initially provided in SpolDB4, and slightly revised in SITVITWEB by the addition of 5 “new rules” for definition of variants within 62 existing lineages/sub-lineages. These include specific signatures for various *M. tuberculosis* complex members, as well as rules defining major lineages/sub-lineages for *M. tuberculosis* stricto sensu, i.e., the Beijing clade, the Central Asian (CAS) clade and two sublineages, the East African-Indian (EAI) clade and nine sublineages, the Haarlem (H) clade and three sublineages, the Latin American-Mediterranean (LAM) clade and 12 sublineages, the “Manu” family and three sublineages, the S clade, the IS*6110*–low-banding X clade and four sublineages, and an ill-defined T clade with five sublineages. Lastly, the recent transmission rate was estimated by the N-1 method [5], according to formula T(c) - N(c)/T(a), where T(c) is the total number of clustered isolates, N(c) is the number of clusters, and T(a) is the total number of isolates.

### Epidemiological, phylogenetical, and statistical analysis

Epidemiological and demographic data collected were analyzed using Epi-Info 3.2.2 software (Centers for Disease Control and Prevention, Atlanta, GA; free download available at http://www.cdc.gov/epiinfo). The statistical analysis was carried out with STATA® 9.0 software (StataCorp, College Station, TX). Patients were divided into 2 groups: clustered patients (infected by strains harboring identical genotypic patterns), versus unclustered patients. The Chi-2, Fischer exact, and Student tests (according to the variables considered) were carried out using univariate analysis in order to test risk factors in function with clustering of the isolates. Odds ratio were calculated with a confidence interval of 95%. A qualitative analysis of the data collected was performed to evaluate the possible ways of infection. The phylogenetical analysis of genotyping data was carried out using “PAUP 4.0” software (available at: http://paup.csit.fsu.edu) and UPGMA (Unweighted Pair Group Method with Arithmetic Averages) algorithm on combined genotyping data.

## Results

### Demographic characteristics, geographic localization, and drug resistance

Epidemiological and demographic data of the 129 patients with a culture-positive, confirmed *M. tuberculosis* infection are summarized in Table 1. The population of TB patients in Guadeloupe is characterized by a male to female sex-ratio of 1.5 with a median age of patients of 40 years. Overall TB principally affected men in the age group 40–59 years (n = 32/77 or 41.6% of all male patients); male patients being older than females (median age of 42 vs. 36 years). More than 1 out of 2 patients was foreign-born (63/129 or 48.8% French vs. 66/129 or 51.2% foreign-born), who had been residing in Guadeloupe for approximately 10 years at the time of diagnostic. The exact place of birth was available for 126/129 patients: 53/126 (42.1%) were born in Haiti, 5/126 (4%) in Dominican Republic and 52/126 (41.3%) in French overseas departments (Guadeloupe n = 43, Martinique n = 1, French Guiana n = 1), or other departments in continental France (n = 7). Foreign-born patients were significantly younger (median age 36 years vs. 50 years for French patients; *p < 0.001*); the proportion of patients below 60 years being 63/66 or 95.5% among foreign-born vs. 40/63 or 63.5% for French patients (*p < 0.001*). Lastly, men were older than females among the foreign-born group: 39 vs. 34 years as compared to 49 vs. 58 years for French nationals (difference not significant statistically). These results corroborate published data from mainland France for French vs. foreign-born patients for the year 2004 [18]. Geographic localization of patients showed that 27/34 cities of Guadeloupe archipelago (including its island dependencies of Saint-Martin, Saint-Barthelemy, Marie-Galante, Desirade, and Les Saintes) declared at least one case of tuberculosis during the period of the study; 6 areas shared 69/116 (59.5%) of the TB cases for which this information was available: Saint-Martin 20/116 (17.2%); Les Abymes 20/116 (17.2%); Le Gosier, 10/116 (8.6%); Pointe-à-Pitre, 8/116 (7.0%); Baillif, 6/116 (5.2%); and Basse-Terre, 5/116 (4.3%). Regarding drug resistance, 10/129 (7.8%) cases were associated to any drug resistance; cumulative resistance to isoniazid and rifampicin (multidrug resistance or MDR) concerned 3/129 (2.3%) cases.

**Table 1:** Demographical and epidemiological data of the 129 tuberculosis patients from Guadeloupe in function of their place of birth

**Parameters studied**		**Total number (%) (n = 129)**	**Foreign-born patients number (%) (n = 66)**	**French patients number (%) (n = 63)**	***p***
Sex					
	male	77 (59.7)	35 (53.0)	42 (66.7)	*NS*
	female	52 (40.3)	31 (47.0)	21 (33.3)	
	unknown	0	0	0	
Age group					
	0-14 years	1 (0.8)	1 (1.5)	0 (0)	*<0.001*
	15-24 years	12 (9.3)	10 (15.2)	2 (3.2)	
	25-39 years	44 (34.1)	26 (39.4)	18 (28.6)	
	40-59 years	46 (35.7)	26 (39.4)	20 (31.7)	
	>60 years	26 (20.2)	3 (4.5)	23 (36.5)	
	unknown	0	0	0	
Employment ^a^					
	no	65 (70.7)	37 (72.5)	28 (68.3)	*NS*
	yes	27 (29.3)	14 (27.5)	13 (31.7)	
	unknown	37	15	22	
Positive HIV status					
	no	77 (75.7)	34 (61.8)	43 (91.5)	*<0.001*
	yes	25 (24.5)	21 (38.2)	4 (8.5)	
	unknown	27	11	16	
Smoker					
	no	76 (80.0)	45 (83.3)	31 (75.6)	*NS*
	yes	19 (20.0)	9 (16.7)	10 (24.4)	
	unknown	34	12	22	
Excessive alcohol user ^b^					
	no	69 (71.9)	47 (85.5)	22 (53.7)	*<0.001*
	yes	27 (28.1)	8 (14.5)	19 (46.3)	
	unknown	33	11	22	
Drug user					
	no	81 (87.1)	50 (96.2)	31 (75.6)	*<0.01*
	yes	12 (12.9)	2 (3.8)	10 (24.4)	
	unknown	36	14	22	
Prior history of active TB					
	no	90 (87.4)	53 (93.0)	37 (80.4)	*NS*
	yes	13 (12.6)	4 (7.0)	9 (19.6)	
	unknown	26	9	17	
Sputum AFB smear result					
	no	38 (29.5)	21 (31.8)	17 (27.0)	*NS*
	yes	91 (70.5)	45 (68.2)	46 (73.0)	
	unknown	0	0	0	
Drug resistance					
	no	119 (92.2)	61 (92.4)	58 (92.1)	*NS*
	yes	10 (7.8)	5 (7.6)	5 (7.9)	
	unknown	0	0	0	
Localization of TB ^c^					
	pulmonary	114 (88.4)	54 (81.8)	60 (95.2)	*<0.02*
	extrapulmonary	15 (11.6)	12 (18.2)	3 (4.8)	
	unknown	0	0	0	
Treatment outcome ^d^					
	completed treatment / cured	63 (55.8)	37 (66.1)	26 (45.6)	*NS*
	died before treatment completed	17 (15.0)	6 (10.7)	11 (19.3)	
	lost to follow up	33 (29.2)	13 (23.2)	20 (35.1)	
	unknown	16	10	6	

^a^ Employment: the “yes” includes patients who answered positively to the question “Do you currently have a regular professional activity?”

^b^ Excessive alcohol user: “yes” concerns patients drinking more than 4 glasses of alcoholic beverages per day.

^c^ Localization of TB: Pulmonary, patients without any extrapulmonary manifestation of TB; Extrapulmonary, patients with extrapulmonary or disseminated TB, as well as with combined manifestations of extrapulmonary and pulmonary TB.

^d^ Treatment outcome: “completed treatment” includes only cured patients.

Abbreviations: *NA* Not Applicable, *NS* Non Significant (significance level below 0.05).

### Access to healthcare and delay to diagnosis

Access to healthcare was defined as the delay comprised between the first symptoms of TB and the date of its clinical suspicion. This interval includes 2 periods: the access time to 1^st^ medical contact which is the delay comprised between the 1^st^ symptoms of TB and the date of 1^st^ medical contact for those symptoms, and the time of clinical suspicion defined as the delay extending from the date of 1^st^ medical contact to the date of clinical suspicion of TB. In the present study, the median access time to 1^st^ medical contact and to clinical suspicion were equal to 16 and 26.5 days respectively leading to a median delay to healthcare of 74.5 days. We observed that patients treated after clinical suspicion presented a significantly longer median delay of access to healthcare than those treated after bacteriological investigations (median delay of 80 vs. 37 days respectively; *p < 0.017*); due to a longer access time to 1^st^ medical contact (median delay of 19 *vs*. 8 days respectively) and a significantly longer delay of clinical suspicion (33.5 *vs*. 10.5 days respectively; *p < 0.03*). Remarkably, in both groups of patients, the diagnostic delay was longer than the access time to the first medical contact. The primary reason for consulting a physician for a first medical contact was persistent cough with fever (median 16 days). TB was suspected at the 1^st^ medical contact for 69/107 (64.5%) patients, 2^nd^ contact for 22/107 (20.6%), patients, 3^rd^ contact for 10/107 (9.3%) patients, and later for 6/107 (5.6%) patients. Patients for whom a presumptive diagnosis of TB was made at the 1st medical contact had a longer delay in consulting the physician after the onset of clinical symptoms (30.5 *vs*. 3.5 days for other patients; *p < 0.0001*). Nonetheless, these were diagnosed for TB significantly faster than the remaining patients (10.5 *vs*. 74 days; *p = 0.025*) most probably due to a more advanced state of the disease; which suggests that efforts targeting early medical diagnosis of TB must be intensified.

### Socioeconomic characteristics

As summarized in Table 1, only 27/92 (29.3%) patients had a regular work (employees n = 13, workmen n = 4, farmers n = 4, craftsmen n = 3, management n = 2, and unspecified job n = 1). The remaining 65/92 (70.7%) patients were jobless, nonetheless 19/65 (29.2%) reported doing small undeclared jobs from time to time. Overall, the TB patients constituted a low income group with an average earning of 824 EUR/month (range, 40 – 5000 EUR). The underprivileged nature of the TB patient group was also reflected by the fact that 18.9% shared a single room with 2 or more persons, and 52.2% used regularly public transport. Regarding addiction, 27/96 (28.1%) were regular alcohol consumers (more than 4 glasses of alcoholic beverages/day), and 19/95 (20.0%) were smokers among whom 10/19 (52.6%) smoked more than a packet of 20 cigarettes/day. Drug consumption was known for 93 patients, and 12/93 (12.9%) declared consuming cannabis and/or crack regularly. Lastly, only 10/91 (11%) patients affirmed having frequent relation to public, although 53/91 patients (58.2%) attended church regularly. During the 2 years preceding the diagnosis of TB, 17/89 (19.1%) patients had carried out 21 trips in 14 French departments (11 French and 6 foreign-born). In general, the French nationals mostly traveled to mainland France (10/11), as opposed to foreign-born patients who traveled within the French departments of Americas (4/6). Despite the highly precarious living conditions of the patient group, 27/89 (30.3%) patients also carried out 42 international travels in 12 countries (mostly to neighboring Caribbean countries). These trips outside the French overseas departments essentially concerned foreign-born patients returning to their homeland, the highest proportion being made up of Haitians (n = 16).

### Clinical characteristics and treatment outcome

Table 1 also describes clinical characteristics of the 129 patients of the study. Data collected showed that extrapulmonary TB was more frequent among the foreign-born group of patients as compared to French nationals (12/66 or 18.2% vs. 3/63 or 4.8%; *p <0.02*). Previous history of TB was known for 103/129 patients; 90 were new cases (87.4%) while 13 patients (14.4%) were previously treated for TB (8 pulmonary, 1 extrapulmonary, 1 pulmonary case associated with a genital form, 3 unknown). Underlying medical conditions were known for 92/129 patients, and revealed that 38.0% (35/92) of the patients were also simultaneously treated for associated diseases (diabetes, prostate cancer, liver cancer, arterial hypertension, asthma). HIV serology was known for 102/129 patients (55 foreign-born and 47 French nationals) with a global TB/HIV co-infection rate of 24.5% (25/102); the latter was significantly higher among foreign-born group (21/55 or 38.2% *vs.* 4/47 or 8.5% for French nationals; *p = 0.001*). This co-infection rate of 24.5% places Guadeloupe largely ahead of metropolitan France with 10% of TB/HIV co-infection for the same time period [19].

Treatment outcome was known for 113/129 patients; 63/113 (55.8%) were cured, 33/113 (29.2%) were lost to follow-up, and 17/113 (15%) died within 4 months after the diagnostic of TB, mostly due to underlying health conditions (Table 1). Among those lost of sight (n = 33), 26 patients followed their initial treatment for an unspecified period of time (median delay of 4 months after the initial phase of treatment in hospital, no information available for 7 patients).

### Genotyping, clustering, and phylogenetical analysis

**Figure 1:**
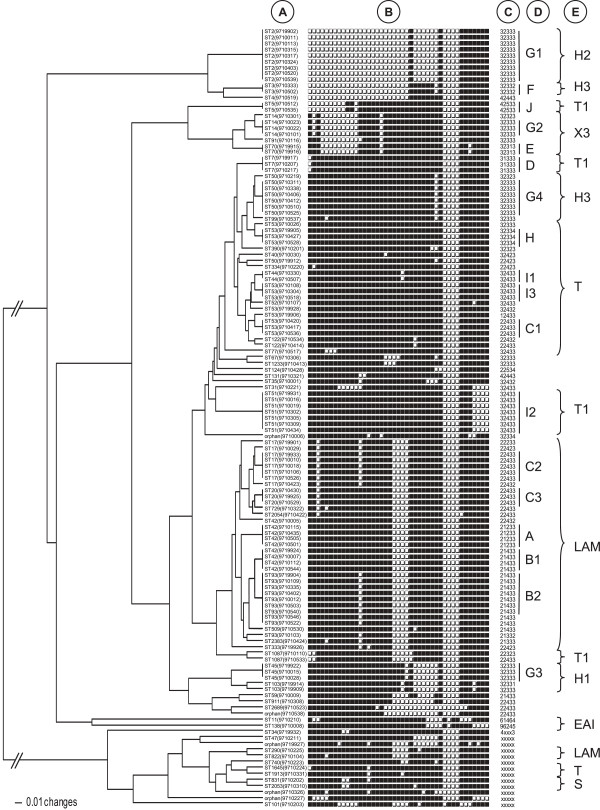
**UPGMA dendrogram obtained through a combined numerical analysis of spoligotypes and VNTR profiles of the 129** ***M. tuberculosis *clinical isolates from Guadeloupe.** The letters in front of the tree correspond to the following information: **A**: SIT numbers of the spoligotype profiles with, in brackets, the strain numbers; **B**: binary spoligotypes; **C**: 5-loci VNTR profiles. The “x” symbol among a profile indicates a missing or uninterpretable result; **D**: clusters numbers; **E**: clades attribution of the spoligotype profiles.

Spoligotyping of the 129 clinical isolates generated 57 different patterns presented in Figure 1 and Additional file 1: Table S1, among which 49 profiles were already present in our database with a SIT number, 3 profiles matched orphan patterns in the database creating new shared-types (SITs 1913, 2383, and 2689), and 5 were not reported earlier and labeled as orphan. A total of 90/129 (69.8%) isolates were grouped into 18 clusters (2 to 12 strains per cluster) while 39/129 (30.2%) were unique in this study. The overall lineage distribution was as follows: LAM (n = 38/129 or 29.5%), ill-defined T family (n = 39/129 or 30.2%), Haarlem (n = 30/129 or 23.3%), X clade (n = 8/129 or 6.2%), S family (n = 3/129 or 2.3%), EAI (n = 2/129 or 1.6%), and AFRI (n = 1/129 or 0.8%).

We also looked in the updated version of the SITVIT2 database for the geographical distribution of all clustered isolates found in Guadeloupe vs. their relative distribution in all the 3 French Departments of the Americas (Guadeloupe, Martinique, and French Guiana), Caribbean, South and central America, as well as the remaining countries worldwide (database interrogation made on April 25^th^ 2013; Table 2). One can observe essentially 5 kinds of situations: (i) ubiquitous spoligotypes present in a very high proportion in the database (SIT42/LAM9, SIT50/H3, SIT53/T1), (ii) patterns that are shared between the 3 French departments, Caribbean, South and Central America and the rest of the world, although in varying proportions (SIT2/H2, SIT3/H3, SIT7/T1, SIT20/LAM1, SIT44/T5, SIT45/H1, SIT51/T1, SIT93/LAM5, SIT122/T1), (iii) patterns with a phylogeographical specificity for South and Central America that are present in Caribbean including Guadeloupe (SIT17/LAM2); (iv) patterns that are shared between Caribbean and the rest of the world but absent in South and Central America (SIT5/T1, SIT70/X3); (v) patterns that show phylogeographical specificity for French Departments in general and Guadeloupe in particular (SIT14/X3, SIT103/H1, SIT1087/T1).

**Table 2:** **Worldwide geographical distribution of major SITs found in Guadeloupe according to the SITVIT2 database (interrogation made on April 25**^**th**^**, 2013)**

**SIT**	**Spoligotype description**	**Lineage**	**Total no. of strains (SITVIT2)**	**Geographical distribution **^**a**^					
				**GLP**	**MTQ**	**GUF**	**Caribbean**	**South and Central America**	**Other countries**
2	□□□□□□□□□□□□□□□□□□□□□□□□■□□□□□□■□□□□■■■■■■■	H2	393	18	5	24	21(CUB), 37(HTI)	35(ARG), 21(BRA), 2(COL), 3(HND), 2(PAN), 1(PRY)	224
3	□□□□□□□□□□□□□□□□□□□□□□□□■■■■■■□■□□□□■■■■■■■	H3	105	4	0	0	3(CUB), 1(HTI)	2(ARG), 7(BRA), 3(COL), 1(SUR), 1(VEN)	83
5	□□□□□□□□□■■□■■■■■■■■■■■■■■■■■■■■□□□□■■■■■■■	T1	30	3	2	4	7(HTI)	0	14
7	□■■■■■■■■■■■■■■■■■■■■■■■■■■■■■■■□□□□■■■■■■■	T1	77	4	0	4	1(BHS), 2(HTI)	1(PER), 2(PRY)	63
14	■□■□□□□□□□□□■■■■■□■■■■■■■■■■■■■■□□□□■■■■■■■	X3	42	27	6	2	0	1(VEN)	6
17	■■□■■■■■■■■■□■■■■■■■□□□□■■■■■■■■□□□□■■■■■■■	LAM2	656	22	4	12	1(CUB), 9(DOM), 22(HTI), 1(TTO)	2(ARG), 184(BRA), 13(COL), 1(PAN), 1(PER), 1(PRY), 179(VEN)	204
20	■■□■■■■■■■■■■■■■■■■■□□□□■■■■■■■■□□□□■■■■■■■	LAM1	811	8	1	20	5(CUB), 26(HTI), 3(JAM)	2(ARG), 147(BRA), 14(COL), 5(PER), 1(PRY), 42(VEN)	537
42	■■■■■■■■■■■■■■■■■■■■□□□□■■■■■■■■□□□□■■■■■■■	LAM9	3282	24	8	31	**Ubiquitous**	**Ubiquitous**	**Ubiquitous**
44	■■■■■■■■■■■■■■■■■■■■■■□■■■■■■■■■□□□□■■■■■■■	T5	210	4	0	6	0	3(ARG), 3(BRA), 1(SUR), 4(VEN)	189
45	■■■■■■■■■■■■■■■■■■■■■■■□■□□□□□□■□□□□■■■■■■■	H1	106	8	14	0	1(HTI), 3(LCA)	9(BRA), 8(COL), 1(PAN)	62
50	■■■■■■■■■■■■■■■■■■■■■■■■■■■■■■□■□□□□■■■■■■■	H3	3330	23	16	88	**Ubiquitous**	**Ubiquitous**	**Ubiquitous**
51	■■■■■■■■■■■■■■■■■■■■■■■■■■■■■■■■□□□□■■■□□□□	T1	279	12	0	13	17(HTI), 3(JAM)	4(ARG), 40(BRA), 1(PER), 10(VEN)	179
53	■■■■■■■■■■■■■■■■■■■■■■■■■■■■■■■■□□□□■■■■■■■	T1	6104	39	25	84	**Ubiquitous**	**Ubiquitous**	**Ubiquitous**
70	■■■□□□□□□□□□■■■■■□■■■■■■■■■■■■■■□□□□■■□■■■■	X3	116	3	0	6	8(BHS), 8(HTI), 1(TTO)	0	90
93	■■■■■■■■■■■■□■■■■■■■□□□□■■■■■■■■□□□□■■■■■■■	LAM5	359	19	0	9	7(DOM), 15(HTI), 1(TTO)	2(ARG), 43(BRA), 2(COL), 17(PER), 6(PRY), 94(VEN)	144
103	■■■■■■■■■■■■■■■■■■■■■■□■■□□□□□□■□□□□■■■□■■■	H1	3	3	0	0	0	0	0
122	■■■■■■■■■■■■■■■■■■■■■■■■■□■■■■■■□□□□■■■■■■■	T1	27	2	0	0	0	1(COL), 1(PAN)	24
1087	□□■■■■■■■■■■■■■■■■■■□□□□□■■■■■■■□□□□■■■■■■■	T1	4	2	0	0	1(HTI)	0	1

^a^ Distribution in the 3 French Departments of Americas, the Caribbean islands, South and Central America, and elsewhere in the world according to the SITIVT2 database. The 3-letters code for Argentina (ARG), Brazil (BRA), Bahamas (BHS), Columbia (COL), Cuba (CUB), Dominican Republic (DOM), French Guiana (GUF), Guadeloupe (GLP), Haiti (HTI), Honduras (HND), Jamaica (JAM), Martinique (MTQ), Panama (PAN), Paraguay (PRY), Peru (PER), Saint Lucia (LCA), Suriname (SUR), Trinidad and Tobago (TTO), and Venezuela (VEN) are according to http://fr.wikipedia.org/wiki/ISO_3166-1.

Genotyping of the 5-loci VNTRs provided complete and/or interpretable results for 116/129 strains (Figure 1) with 15 clusters (n = 106 isolates, 2–27 strains/cluster). Note that 9 of the 10 unclustered strains (with the exception of pattern 96245) matched an existing VIT number in the database, and no VIT numbers were newly created due to a newly found pattern or a match with preexisting orphan in the database.

The combination of spoligotypes and VNTRs revealed 76 distinct profiles for the 129 isolates: 58 corresponded to unique vs. 18 for clustered strains (n = 71, 2–9 isolates/cluster; labeled as A, B1, B2, C1, C2, C3, D, E, F, G1, G2, G3, G4, H, I1, I2, I3, and J in Figure 1). When epidemiological data were analyzed in combination with clustering, we observed that mean age of patients among the 18 clusters varied from 27 (cluster I1, n = 2) to 59 years old (cluster G2, n = 3), and that the male to female sex ratio varied from 0 (cluster E, n = 2) to 3 (cluster A, n = 4); with 4 clusters composed exclusively of male patients (clusters C1 n = 3; D n = 3; G2 n = 3; J n = 2). Regarding drug resistance, 3 clusters contained 1 MDR strain each (clusters J, C2, and B2; the MDR strain of C2 was simultaneously resistant to INH, RIF, EMB while that of cluster J to INH, RIF, EMB, SM). Furthermore, 1 cluster contained 2 monoresistant strains (to SM and INH, cluster G4); while 2 others contained 1 monoresistant strain each to INH (cluster C3) and to SM (cluster H).

It should be underlined that the cluster I1 composed of the youngest patients, did not contain any drug resistant strains, and that the 3 patients infected with MDR strains were simultaneously HIV-positive. A comparison with the SITVIT2 database showed that the combined spoligotyping + 5-loci VNTR profiles of the 6 clusters including resistant strains, matched mainly strains from Haiti. For example, 9/10 strains in the database sharing cluster J pattern (SIT5/VIT193) were isolated from Haitian patients. Similarly, 14/16 patients with cluster B2 pattern (SIT93/VIT3); 20/24 strains with cluster C2 pattern (SIT17/VIT10), 26/36 strains with cluster C3 pattern (SIT20/VIT10), and 34/84 of strains with cluster G4 pattern (SIT50/VIT18), were also isolated from Haitian patients in the database. Unfortunately, drug resistance profiles were not systematically available in the database, making it impossible to definitely link the genotypes and drug resistance of isolates actively circulating between Haiti and Guadeloupe.

### Retrospective evaluation of clustered isolates by 12-loci MIRU typing

Because during the time course of this 7 years prospective study, the TB molecular typing methodology evolved with the introduction of 12-loci MIRU typing [13,14], we decided to retrospectively genotype a subset of 44 *M. tuberculosis* strains clustered by the combination of spoligotyping and 5-loci VNTRs. The results obtained (Table 3) showed that 26/44 (59.1%) strains remained clustered vs. 18/44 (40.9%) being unique by the combination of the 3 methods. This allowed decreasing the clustering rate and recent transmission rates in this subset from 55% and 41.1% respectively, to 25.5% and 16.7% respectively.

**Table 3:** **Retrospective 12-loci MIRU analysis of a subset of 44** ***M. tuberculosis *strains initially clustered by using the combination of spoligotyping with 5-loci VNTRs**

**Cluster**	**SIT**	**Spoligotype**	**VNTR**	**VIT**	**12-loci MIRU**	**MIT**	**Nb of strains**
F (n = 2)	3	□□□□□□□□□□□□□□□□□□□□□□□□■■■■■■□■□□□□■■■■■■■	32332	17	224313153223	181	1
					225313153223	810	1
J (n = 2)	5	□□□□□□□□□■■□■■■■■■■■■■■■■■■■■■■■□□□□■■■■■■■	42533	193	223325153322	15	2
G2 (n = 3)	14	■□■□□□□□□□□□■■■■■□■■■■■■■■■■■■■■□□□□■■■■■■■	32333	18	224325133324	122	1
					224325133325	28	1
					224325133326	1042	1
C2 (n = 5)	17	■■□■■■■■■■■■□■■■■■■■□□□□■■■■■■■■□□□□■■■■■■■	22433	10	224226163321	26	4
					Not Amplified		1
C3 (n = 3)	20	■■□■■■■■■■■■■■■■■■■■□□□□■■■■■■■■□□□□■■■■■■■	22433	10	223226163321	307	2
					223226193321	1048	1
A (n = 4)	42	■■■■■■■■■■■■■■■■■■■■□□□□■■■■■■■■□□□□■■■■■■■	21233	2	224226143321	738	1
					224226153321	25	3
B1 (n = 4)	42	■■■■■■■■■■■■■■■■■■■■□□□□■■■■■■■■□□□□■■■■■■■	21433	3	224226153321	25	3
					233126142321	1539	1
I1 (n = 2)	44	■■■■■■■■■■■■■■■■■■■■■■□■■■■■■■■■□□□□■■■■■■■	32433	23	223326153323	236	2
G4 (n = 6)	50	■■■■■■■■■■■■■■■■■■■■■■■■■■■■■■□■□□□□■■■■■■■	32333	18	224325153321	161	1
					225313153322	184	1
					225313153323	42	3
					225323153323	43	1
H (n = 3)	53	■■■■■■■■■■■■■■■■■■■■■■■■■■■■■■■■□□□□■■■■■■■	32334	19	227325163423	802	1
					228225163423	806	1
					228325163423	415	1
C1 (n = 3)	53	■■■■■■■■■■■■■■■■■■■■■■■■■■■■■■■■□□□□■■■■■■■	22433	10	222325153324	146	1
					223225153322	296	1
					223425143322	160	1
B2 (n = 7)	93	■■■■■■■■■■■■□■■■■■■■□□□□■■■■■■■■□□□□■■■■■■■	21433	3	224226143321	738	2
					224226153321	25	5

### Contact tracing investigations and risk factor analysis

Rebuilding the ways of infection of TB cases due to a recent infection was carried out by cross analyzing genotyping data in conjunction with results of contact tracing investigations, as well as data collected through individual interviews. Contact tracing alone suggested a potential common source of infection for 6 patients of the study, which in our opinion is underestimated since only 54% of the TB cases diagnosed could be effectively covered by investigations among patient’s entourage. Genotyping results combined with epidemiologic investigations underlined 23 TB cases categorized as recent transmission. Finally, the consideration of all the 3 parameters confirmed active transmission of TB for 24/129 (18.6%) of cases.

A Univariate analysis of risk factors was performed in function to the clustering of *M. tuberculosis* isolates and available demographic, epidemiologic, clinical, and socioeconomic data. However, the results summarized in Table 4 showed that none of the risk factors tested could be significantly associated with active transmission of TB. Only the factor “age of patients below 60 years” was close to the statistical limit of significance (*p = 0.06*).

**Table 4:** **Univariate analysis of risk factors in relation to the prospective genotype based clustering analysis of 129** ***M. tuberculosis *isolates following spoligotyping and 5-loci VNTRs (clustered, n = 71 vs. unclustered, n = 58)**

**Risk factors**	**No. of patients (%)**		**Odds ratio (95% CI)**	***p *value**
	**Clustered**	**Unclustered**		
Male sex	40 (56.3)	37 (63.8)	0.73 [0.34-1.58]	0.40
Low incomes (< 465 €)	21 (29.6)	14 (24.1)	2.03 [0.73-5.68]	0.13
Alcohol abuse ^a^	15 (21.1)	12 (20.7)	0.73 [0.25-2.15]	0.50
Pulmonary localization of TB	64 (90.1)	50 (86.2)	1.46 [0.44-4.87]	0.50
Age < 60 years	61 (85.9)	42 (72.4)	2.32 [0.89-6.16]	0.06
HIV positive	16 (22.5)	9 (15.5)	1.41 [0.51-3.96]	0.50
Foreign born	36 (50.7)	30 (51.7)	0.96 [0.48-1.92]	0.90
Underlying conditions ^b^	30 (42.3)	28 (48.3)	0.78 [0.37-1.67]	0.50
Previous medical problems ^c^	16 (22.5)	19 (32.8)	0.78 [0.25-2.41]	0.60
Diagnosis delay ^d^ > 26.5 j	44 (62.0)	43 (74.1)	0.57 [0.25-1.29]	0.14
Hospitalization	18 (25.4)	10 (17.2)	1.80 [0.64-5.13]	0.20

^a^ patients drinking more than 4 glasses of alcoholic beverages per day.

^b^ homeless or living in shelters.

^c^ other than preliminary tuberculosis.

^d^ median delay between the first medical contact for tuberculosis symptoms and clinical suspicion of tuberculosis.

## Discussion

This study is the first prospective epidemiological and molecular study of TB transmission in the French overseas department of Guadeloupe. When compared to TB epidemiology in mainland France, the most striking difference was the significantly higher proportion of TB/HIV co-infected patients in Guadeloupe (24.5% *vs.* 10% respectively; [19]). However, an almost similar proportion of drug-resistance and TB incidence were observed in 2005: the proportion of drug resistant isolates being 7.8% vs. 8.8%, respectively, in Guadeloupe and continental France [19], and an incidence of 8.1 vs. 8.9 cases per 100,000 inhabitants [20]. Similarly to mainland France, men represented around 60% of TB patients in Guadeloupe a trend accentuated for the age group 40–60 years in mainland France (68% of TB patients) and 40–59 years in Guadeloupe (71.1% of TB patients [20]). Lastly, TB in Guadeloupe was characterized by immigration with 51.2% of foreign-born patients in our study, which is slightly higher than 48% in mainland France in 2005, but significantly lower than the 69% of foreign born patients observed in French Guyana (185/268; *p < 0.001*; [20,21]). Immigrants in Guadeloupe mainly originated high TB incidence and AIDS burden surrounding Caribbean islands of Haiti and Dominican Republic, with reported TB incidence in 2005 of 305 and 91 cases/100 000 inhabitants [22], which might explain the relatively high rate of TB/HIV coinfected patients among foreign born patients in our study (38.2% *vs*. 8.5% for French patients; *p = 0.001*).

The present study was meant to specifically evaluate the use of genotyping to quantify recent TB transmission with a focus on risk factors and access to health care during 1999–2005; and was followed by a study on evolution of TB cases during 2006–2011 [23]. We therefore attempted to compare TB evolution during these 2 time periods. As summarized in Table 5, one may notice a statistically significant decrease during the period 2006–2011 for 4 out of 9 parameters compared, namely, the proportion of foreign-born patients; patients lost of site; excessive alcohol users; drug addicts. The decrease in the proportion of foreign-born patients from 51.2% vs. 38.3% may be linked to the decrease in the proportion of migrants observed in Guadeloupe from 6.5% to 4.7% in 2010 [24,25]. It may be noted that the decrease among lost of site is statistically significant both among foreign-born and French patients, while the decrease in excessive alcohol users and drug addicts was limited to French patients only. Regarding the 5 parameters that did not change significantly (Table 5), it might be worthwhile to comment on trends for TB/HIV coinfection which remained stable between the periods 1999–2005 and 2006–2011 (24.5% to 26.6%), despite a slight but non-significant increase among both French-born (8.5% to 13.9%) and foreign born patients (38.2% vs. 46.9%). However, considering the reported decrease in the incidence of HIV/AIDS infection in Guadeloupe from 20 to 11.7 cases/100 000 inhabitant between 2003 and 2009 [26,27], this observation might be explained on the basis of continued influx of a varying proportion of migrant workers from poor surrounding countries such as Haiti and Dominican Republic, where both TB and HIV/AIDS remain highly endemic [1]. This assumption is corroborated by the fact that the proportion of extrapulmonary TB increased in Guadeloupe from 11.6% in 1999–2005 to 15.6% in 2006–2011 (Table 5). In our study, foreign-born patients were more likely to be TB/HIV co-infected and also showed a higher proportion of extra pulmonary disease. These observations are likely not independent, although stratified analysis was not performed to be conclusive.

**Table 5:** **Evolution of epidemiological and clinical data concerning locally born and foreign born populations of TB patients in Guadeloupe between 1999–2005 (this study) and 2006–2011 (Cadelis et al., 2012 **[23]**)**

**Parameters studied**	**Total population of patients**			**Foreign born**			**Locally born**		
	**1999-2005 (n = 129)**	**2006-2011 (n = 128)**	***p***	**1999-2005 (n = 66)**	**2006-2011 (n = 49)**	***p***	**1999-2005 (n = 63)**	**2006-2011 (n = 79)**	***p***
Foreign born	66 (51.2)	49 (38.3)	<0.05	NA	NA				
Male	77 (59.7)	80 (62.5)	NS	31 (47.0)	24 (49.0)	NS	42 (66.7)	56 (70.9)	NS
Prior history of active TB	13 (12.6) ^(a)^	25 (19.5)	NS	4 (7.0) ^(g)^	8 (16.3)	NS	9 (19.6) ^(l)^	17 (21.5)	NS
Extrapulmonary TB	15 (11.7)	20 (15.6)	NS	12 (18.2)	12 (24.5)	NS	3 (4.8)	8 (10.1)	NS
HIV+	25 (24.5) ^(b)^	34 (26.6)	NS	21 (38.2) ^(h)^	23 (46.9)	NS	4 (8.5) ^(m)^	11 (13.9)	NS
Lost of sight	33 (29.2) ^(c)^	8 (6.3)	<0.001	13 (23.2) ^(i)^	1 (2.0)	<0.01	20 (35.1) ^(n)^	7 (8.9)	<0.001
Excessive alcohol user	27 (28.1) ^(d)^	22 (17.2)	0.05	8 (14.5) ^(h)^	3 (6.1)	NS	19 (46.3) ^(o)^	19 (24.1)	<0.025
Smoker	19 (20) ^(e)^	30 (23.4)	NS	9 (16.7) ^(j)^	7 (14.3)	NS	10 (24.4) ^(o)^	23 (29.1)	NS
Drug user	12 (12.9) ^(f)^	6 (4.7)	<0.05	2 (3.9) ^(k)^	1 (2.0)	NS	10 (24.4) ^(o)^	5 (6.3)	<0.01

– Abbreviations: *NA* Not applicable.

– For Total population of patients: Information available for (a) 103/129 cases, (b) 102/129 cases, (c) 113/129 cases, (d) 96/129 cases,.

(e) 95/129 cases, (f) 93/129 cases.

– For foreign-born patients: information available for (g) 57/66 cases, (h) 55/66 cases, (i) 56/66 cases, (j) 54/66 cases, (k) 52/66 cases.

– For French-born patients: information available for (l) 46/63 cases, (m) 47/63 cases, (n) 57/63 cases, (o) 41/63 cases.

Regarding geographical mapping of TB cases, 5 out of 6 cities sharing 60% of TB burden in our study were characterized by higher population densities than the mean reported for Guadeloupe, and 3 out of 6 were also characterized by a higher proportion of underprivileged populations (more than 12.6% [28,29]). Furthermore, TB patients in our study were characterized by a very high rate of unemployment (70.7%; Table 1) and a below average income close to the official limit of poverty in France (monthly income of 824 EUR vs. 812 EUR/month in 2001; [29,30]).

Our results also provided with a first insight into the management of TB in Guadeloupe, and revealed that 38/107 (35.5%) of patients for which information was available, consulted more than one physician before being diagnosed for TB. Interestingly, the management of patients improved in the period 2006–2011 [23] with a median delay of 55 days between the 1^st^ symptoms and clinical suspicion of TB instead of 74.5 days in 1999–2005; as well as the delay for clinical suspicion of TB after a 1^st^ medical consultation (18 days vs. 26.5 days). Comparison of foreign-born vs. French patients did not reveal significant differences in access to healthcare. Regarding treatment outcome, only 55.8% of patients were cured in our study since 15.0% of them deceased, and 29.2% were lost to follow up. These relatively mediocre TB management indicators as compared to Europe (74% treatment success rate, 7% deceased, 12% lost to follow up; [31]), may indirectly be linked to the lack of awareness among the patient population in Guadeloupe, which is characterized by poor living conditions underlined earlier.

As far as feasibility of molecular typing in a prospective study is concerned, our results demonstrated that PCR-based spoligotyping not only correctly identified potential “outbreak” strains, but also allowed a fine characterization of the phylogeographical specificity of certain clones, e.g., SIT14/X3, SIT103/H1, and SIT1087/T1 (Table 2). The 2^nd^-line genotyping using 5-loci VNTRs allowed reducing the overestimation of clustering by spoligotyping [32]. However, it was apparently not enough discriminatory since 12-loci MIRUs performed retrospectively allowed to reduce both the clustering and recent transmission rates (Table 3). This observation argues for the use of spoligotyping with extended MIRU typing formats. In our study, mixed clusters involving both foreign-born and French patients concerned 11/18 or 61% of clusters by spoligotyping and 5-loci VNTRs; however after retrospective 12-loci MIRU typing their number was reduced to 5 mixed clusters containing a total of 18 cases. Thus the suggested minimal requirement based on our study is 12-loci MIRUs, although the optimal format for molecular epidemiological investigations today is a new 15-loci format [33,34]. Nonetheless, seeing the very low recent transmission rates found in our study, the future investigations should be extended to the sister island of Martinique, as well as French Guiana – since important migratory flows and exchanges exist between Guadeloupe and the 2 other French departments of the Americas. This might help detect both migratory flows, as well as direct and casual contacts, leading to the ongoing TB epidemic.

Last but not least, thanks to the comparison with an updated SITVIT2 database, we were able to conclude on the phylogeographical specificity of the circulating *M. tuberculosis* clones in Guadeloupe and surrounding regions. Most of this data commented earlier in the text clearly shows the predominance of evolutionary recent Euro-American group of isolates [16,17] in our study. However, LAM, Haarlem and T lineages predominated in Guadeloupe, while the X lineage strains of suspected Anglo-Saxon descent [16,17,35] were present to a smaller extent. Thus the bulk of TB transmission in Guadeloupe may be attributed to TB lineages shared with neighboring areas in the Caribbean and South and Central America [21,36]. Nonetheless, we further identified some clones such as SIT14/X3, SIT103/H1, and SIT1087/T1 (Table 2), which are highly specific for Guadeloupe. We now hope to extend a detailed molecular epidemiological study on the *M. tuberculosis* isolates from the 3 French departments isolated in 2006–2012 so as to see the evolution of above mentioned genotypes in time and space. Ultimate comparison of clusters retained after 12- and 15-loci MIRUs should be done using 24-loci MIRU-VNTRs [34] followed by the whole genome sequencing [37] of specific clones, in order to shed light on epidemiological and phylogenetical links between strains involved in active transmission, as well as to quantify how much *M. tuberculosis* isolates vary at the genomic level between epidemiologically linked patient clusters [38].

## Conclusions

The present prospective genotyping study aimed to investigate TB transmission during a 7 year period in Guadeloupe, as well as to study the access to health care and follow-up of the TB patients diagnosed. The results obtained underline that the ongoing TB transmission in this insular, low TB-incidence setting of Guadeloupe was characterized by: (i) a significant proportion of imported cases of the disease from neighboring islands; (ii) a significantly higher TB/HIV coinfection among foreign-born cases; and, (iii) a higher proportion of cases affecting older age-group among French patients due to reactivation. Last but not least, this study also emphasized the need for universal typing using spoligotyping and 15-loci MIRUs in future prospective studies in Guadeloupe.

## Competing interests

The authors declare that they have no competing interests.

## Author’s contributions

Under a tripartite collaborative agreement, NR, MT, PC participated in the design and follow-up of the study. NR supervised the handling of pathological specimen, *M. tuberculosis* identification, drug-susceptibility testing, and genotyping of the strains. AA and ML collected epidemiological data; SF and SC interpreted the data; and SF performed statistical analysis. SF, SC, NR wrote the initial institutional report in French (http://www.pasteur-guadeloupe.fr/tb/projects/tuberculose.pdf). JM performed retrospective molecular typing using 12-loci MIRUs and performed SITVIT2 database analysis. NR, SF, JM wrote the present manuscript All authors read and approved the final manuscript.

## Pre-publication history

The pre-publication history for this paper can be accessed here:

http://www.biomedcentral.com/1471-2334/13/364/prepub

## Supplementary Material

Additional file 1: Table S1 Description of 57 spoligotype patterns from 129 *M. tuberculosis* clinical isolates in Guadeloupe, followed by a comparison with the SITVIT2 database (interrogation made on April 25th 2013). Click here for file
