# Supplementary material for: Use of genotyping based clustering to quantify recent tuberculosis transmission in Guadeloupe during a seven years period: analysis of risk factors and access to health care
Source: BMC Infect Dis. 2013 Aug 2;13:364. doi: 10.1186/1471-2334-13-364 (PMC3750484; doi:10.1186/1471-2334-13-364)
Supplement: Additional file 1: Table S1 — Description of 57 spoligotype patterns from 129 M. tuberculosis clinical isolates in Guadeloupe, followed by a comparison with the SITVIT2 database (interrogation made on April 25th 2013). [file 1471-2334-13-364-S1.pdf]

Supplemental Table S1. Description of 57 spoligotype patterns from 129 *M. tuberculosis* clinical isolates in Guadeloupe, followed by a comparison with the SITVIT2 database (interrogation made on April 25th 2013).

SIT[a]	Spoligotype Description	Number (%) of strains in study	% in SITVIT2	% in study vs. database	Clade[b]
2	□□□□□□□□□□□□□□□□□□□□□□□□■□□□□□□■□□□□■■■■■■■	9 (6.98)	0.51	2.29	H2
3	□□□□□□□□□□□□□□□□□□□□□□□□■■■■■■□■□□□□■■■■■■■	2 (1.55)	0.14	1.9	H3
4	□□□□□□□□□□□□□□□□□□□□□□□□■■■■■■■■□□□□■■■■■■■	1 (0.78)	0.47	0.28	Unk
5	□□□□□□□□□■■□■■■■■■■■■■■■■■■■■■■■□□□□■■■■■■■	2 (1.55)	0.04	6.67	T1
7	□■■■■■■■■■■■■■■■■■■■■■■■■■■■■■■■□□□□■■■■■■■	3 (2.33)	0.10	3.9	T1
11	■□□■■■■■■■■■■■■■■■■■■■■■■■■■□□□□■□■■□□□■■■■	1 (0.78)	0.80	0.16	EAI3-IND
14	■□■□□□□□□□□□■■■■■□■■■■■■■■■■■■■■□□□□■■■■■■■	4 (3.10)	0.05	9.52	X3
17	■■□■■■■■■■■■□■■■■■■■□□□□■■■■■■■■□□□□■■■■■■■	8 (6.20)	0.86	1.22	LAM2
20	■■□■■■■■■■■■■■■■■■■■□□□□■■■■■■■■□□□□■■■■■■■	3 (2.33)	1.06	0.37	LAM1
31	■■■■■■■□□□□□□■■■■■■■■■■■■■■■■■■■□□□□■■■□□□□	1 (0.78)	0.10	1.3	T
34	■■■■■■■■□□■■■■■■■■■■■■■■■■■■■■■■□□□□■■■■■■■	1 (0.78)	1.09	0.12	S
35	■■■■■■■■■■■■□■■■■■■■■■■■■■■■□□□■□□□□■■■■■■■	1 (0.78)	0.18	0.71	H4 (Ural-1)
40	■■■■■■■■■■■■■■■■■■□■■■■■■■■■■■■■■□□□□■■■■■■■	1 (0.78)	0.21	0.64	T4
42	■■■■■■■■■■■■■■■■■■■■□□□□■■■■■■■■□□□□■■■■■■■	9 (6.98)	4.30	0.27	LAM9
44	■■■■■■■■■■■■■■■■■■■■■■□■■■■■■■■■■□□□□■■■■■■■	2 (1.55)	0.27	0.95	T5
45	■■■■■■■■■■■■■■■■■■■■■■■□■□□□□□□■□□□□■■■■■■■	3 (2.33)	0.14	2.83	H1
47	■■■■■■■■■■■■■■■■■■■■■■■■■□□□□□□■□□□□■■■■■■■	1 (0.78)	1.96	0.07	H1
50	■■■■■■■■■■■■■■■■■■■■■■■■■■■■■■□■□□□□■■■■■■■	8 (6.20)	4.36	0.24	H3
51	■■■■■■■■■■■■■■■■■■■■■■■■■■■■■■■■□□□□■■■□□□□	7 (5.43)	0.37	2.51	T1
52	■■■■■■■■■■■■■■■■■■■■■■■■■■■■■■■■□□□□■■■□■■■	1 (0.78)	1.18	0.11	T2
53	■■■■■■■■■■■■■■■■■■■■■■■■■■■■■■■■□□□□■■■■■■■	12 (9.30)	7.99	0.2	T1
59	■■■■■■■■■■■■■■■■■■■■□□□□■■□□□□■■□□□□■■■■■■■	1 (0.78)	0.60	0.22	LAM11-ZWE
67	■■■■■■■■■■■■■■■■■■□□□□■■■■■■■■□■□□□□■■■■■■■	1 (0.78)	0.09	1.39	H3
70	■■■□□□□□□□□□■■■■■□■■■■■■■■■■■■■■□□□□■■□■■■■	2 (1.55)	0.15	1.72	X3
77	■■■■□□□■■■■■■■■■■■■■■■■■■■■■■■■■□□□□■■■■■■■	1 (0.78)	0.03	4.76	T1
91	■■■□□□□□□□□□□■■■■□■■■■■■■■■■■■■■□□□□■■■■■■■	1 (0.78)	0.33	0.4	X3
93	■■■■■■■■■■■■□■■■■■■■□□□□■■■■■■■■□□□□■■■■■■■	10 (7.75)	0.47	2.79	LAM5
99	■■■■□■■■■■■■■■■■■■■■■■■■■■■■■□■□□□□■■■■■■■	1 (0.78)	0.10	1.33	H3
101	■■■■■■■□□□□□■■■■■■■■□□□□■■□■■■■■□■■■□□□■■■■	1 (0.78)	0.03	4.35	AFRI2
103	■■■■■■■■■■■■■■■■■■■■■■□■■□□□□□□■□□□□■■■□■■■	2 (1.55)	0.00	66.67	H1
122	■■■■■■■■■■■■■■■■■■■■■■■■■□■■■■■■□□□□■■■■■■■	2 (1.55)	0.04	7.41	T1
124	■■■■■■■■■■■■■■■■■■■■■■■■■■■■■■□□□□□□■■■■■■■	1 (0.78)	0.09	1.52	Unk
131	■■■■■■■■■■■■□□■■■■■■■■■■■■■■■■■■□□□□■■■■■■■	1 (0.78)	0.14	0.92	T1
138	■■■■■■■■■■■■■■■■■■■■■■■■■■■■□□□□■□■■■■■□□□□	1 (0.78)	0.16	0.81	EAI5
290	■■■■■■■■■■■■■■■■■■■■□□□□■■□■■■■■□□□□■■■■■■■	1 (0.78)	0.04	3.33	LAM8
333	■■■■■■■■■■■■□■□■■■■■□□□□■■■■■■■■□□□□■■■■■■■	1 (0.78)	0.01	9.09	LAM5
334	■□■■■■■■■■■■■■■■■■■■■■■■■■■■■■■■□□□□■■■■■■■	1 (0.78)	0.12	1.05	T1
390	■■■■■■■■■■■■■■■■■■■■■■■■■■■■■□□■□□□□■■■■■■■	1 (0.78)	0.04	3.23	H3
509	■■■■■■■■■■■■■■■■■■■■□□□□■□■■■■■■□□□□■■■■■■■	1 (0.78)	0.02	8.33	LAM9
729	■■□■□■■■■■■■■■■■■■■■□□□□■■■■■■■■□□□□■■■■■■■	1 (0.78)	0.01	16.67	LAM1
740	■■■■■■■■■■■■■■■■■■■■■■□□■■■■■■□■□□□□■■■■■■■	1 (0.78)	0.02	7.69	H3
822	■■■■□■■■■■■■■■■■■■■■□□□□■■■■■■■■□□□□■■■■■■■	1 (0.78)	0.03	4.76	LAM9
831	■■■■■■■■□□■■■■□■■■■■■■■■■■■■■■■■□□□□■■■■■■■	1 (0.78)	0.02	7.69	S
911	■■■■■■■■■■■■■■■■■■■■□□□□□□□□□□■■□□□□■■■■■■■	1 (0.78)	0.04	2.94	Unk
1087	□□■■■■■■■■■■■■■■■■■■□□□□□■■■■■■■□□□□■■■■■■■	2 (1.55)	0.01	50.00	T1
1233	■■■■■■■■■■■■■■■■■■□□□■■■■■■■■■■■□□□□■■■■■■■	1 (0.78)	0.01	12.5	T1
1645	□■■■■■■■■■■■■■□■■■■■■■■■■■■■■■■■□□□□■■■■■■■	1 (0.78)	0.01	25.00	T1
2053	■■■■■■■■□□■■■■■■■■■■■■■■■□■■■■■■□□□□■■■■■■■	1 (0.78)	0.01	25.00	S
2054	■■□■■■■■■■■■■■■■■■■■□□□□■■■■■■■■□□□□□■■■■■■	1 (0.78)	0.01	25.00	LAM
1913*	■■■■■■■■■■■■■■□■■■■■■■□■■■■■■■■■□□□□■■■■■■■	1 (0.78)	New SIT	New SIT	T5
2383*	■■■■□■■■■■■■□■■■■■■■□□□□■■■■■■■■□□□□■■■■■■■	1 (0.78)	New SIT	New SIT	LAM5
2689*	■■■■■■■■■■■■■■■■□■□□□□□□□□□□□□□□□□□□□□■■■■■	1 (0.78)	New SIT	New SIT	Unk
orphan1	■■■■■■■■■■■■■■□■■□■■■■■■■■■■■■■■□□□□■■□□■■■	1 (0.78)	-	-	X1
orphan2	■■■■■■■□■■■■■■□■■■■■□□□□■□□□□□□■□□□□■■□■□■■	1 (0.78)	-	-	Unk
orphan3	■□□□□■■■■■■■■■■■■■■■■■■■■■■■■■■■□□□□■■■□□□□	1 (0.78)	-	-	Unk
orphan4	■■■■□■■■■■■■■■□■■■■■■■■■■■■□■■□□□□□□■■■■■■■	1 (0.78)	-	-	Unk
orphan5	■■■■■■■■■■■■■■■■■■□□□□□□□□□□□□□■□□□□■■■■■■■	1 (0.78)	-	-	Unk

[a] In this study, 57 different spoligotypes patterns were obtained from 129 *M. tuberculosis* clinical isolates; 90/129 (69.8%) were clustered (18 clusters, 2-12 strains/cluster), and 39/129 (30.2%) were unclustered. A total of 49 SITs containing 121 isolates matched a pre-existing shared type in the SITVIT2 database, whereas 3 SITs (containing 3 isolates) were newly-created after a match with an orphan in the database (showed by an asterisk; SIT1913, SIT2383, SIT2689). Unique strains that did not match any spoligotype pattern in the SITVIT2 database are designed as "orphan" (n=5).

[b] Clade designations according to SITVIT2 using revised SITVITWEB rules; Unk: Unknown pattern within any of the major clades described in SITVIT2 database. Note that SIT35/H4 pattern in SITVITWEB was recently reclassified as Ural-1.
